# Will Ottawa Aid the Movement?

**Published:** 1902-12

**Authors:** 


### WILL OTTAWA AID THE MOVEMENT?

THE announcement that the Grand Rapids, Michigan, Veterinary College will next year inaugurate a three-years' course; the fact that the Cincinnati Veterinary College has reorganized under a three-years' course of six months each, and that the San Francisco Veterinary College, recently reorganized, has established the same curriculum, leaves but two schools of any importance under the ban of requirements of the A. V. M. A. The decision to meet in Ottawa in 1903, for the Convention of the A. V. M. A. amidst the great body of graduates of the Ontario Veterinary College, we may hope to have a benign influence over their alma mater and a fitting tribute and trophy for the first meeting held outside the borders of the United States. If this one strong school should move in this direction there would no longer be any place for the remaining two-year schools at Indianapolis, Kansas City, and Nashville.

---

THE death of Dr. Junius H. Wattles, Sr., ends the career of one of the most aggressive factors in the field of veterinary college work in the central West. Dr. Wattles had organized two veterinary colleges in Kansas City, Mo., and maintained each of them under very trying circumstances and at times discouraging conditions. As individual enterprises and private institutions he continued them under short-term curriculums in order to maintain them financially, and in this way ostracised himself and his schools from the recognition of the profession and the support of the associations. An aggressive foe, untiring in energy and resourceful in every-day affairs, he was potential in his State and city in preventing legislation that would injure the interests of the schools he maintained.

THE amalgamation of *The Veterinarian* and *The Journal of Comparative Pathology and Therapeutics* is a movement in English veterinary journalism of much import to the profession. The strength of its editorial direction will add much to the worth of this periodical, and, we trust, add a stronger support to English veterinary journalism.

---

THE JOURNAL in closing the year 1902 desires to sincerely thank its readers for the patient indulgence they have accorded the editor. The death of the senior editor, the retirement of another of the staff, and the impaired health for several months of the remaining editor, delayed the issues of the JOURNAL to an unusual extent. With the new year and the added strength to our editorial staff we hope to maintain the same good-will and support of those who have long given to the JOURNAL a loyal support.

